# Global freshwater distribution of *Telonemia* protists

**DOI:** 10.1093/ismejo/wrae177

**Published:** 2024-09-20

**Authors:** Roudaina Boukheloua, Indranil Mukherjee, Hongjae Park, Karel Šimek, Vojtěch Kasalický, Maxon Ngochera, Hans-Peter Grossart, Antonio Picazo-Mozo, Antonio Camacho, Pedro J Cabello-Yeves, Francisco Rodriguez-Valera, Cristiana Callieri, Adrian-Stefan Andrei, Jakob Pernthaler, Thomas Posch, Albin Alfreider, Ruben Sommaruga, Martin W Hahn, Bettina Sonntag, Purificación López-García, David Moreira, Ludwig Jardillier, Cécile Lepère, Corinne Biderre-Petit, Anna Bednarska, Mirosław Ślusarczyk, Viktor R Tóth, Horia L Banciu, Konstantinos Kormas, Sandi Orlić, Danijela Šantić, Gerard Muyzer, Daniel P R Herlemann, Helen Tammert, Stefan Bertilsson, Silke Langenheder, Thomas Zechmeister, Nico Salmaso, Nicola Storelli, Camilla Capelli, Fabio Lepori, Vojtěch Lanta, Helena Henriques Vieira, Fran Kostanjšek, Kateřina Kabeláčová, Maria-Cecilia Chiriac, Markus Haber, Tanja Shabarova, Clafy Fernandes, Pavel Rychtecký, Petr Znachor, Tiberiu Szőke-Nagy, Paul Layoun, Hon Lun Wong, Vinicius Silva Kavagutti, Paul-Adrian Bulzu, Michaela M Salcher, Kasia Piwosz, Rohit Ghai

**Affiliations:** Department of Aquatic Microbial Ecology, Institute of Hydrobiology, Biology Centre of the Czech Academy of Sciences, 37005, České Budějovice, Czech Republic; Faculty of Science, University of South Bohemia, 37005, České Budějovice, Czech Republic; Department of Aquatic Microbial Ecology, Institute of Hydrobiology, Biology Centre of the Czech Academy of Sciences, 37005, České Budějovice, Czech Republic; Department of Aquatic Microbial Ecology, Institute of Hydrobiology, Biology Centre of the Czech Academy of Sciences, 37005, České Budějovice, Czech Republic; Department of Aquatic Microbial Ecology, Institute of Hydrobiology, Biology Centre of the Czech Academy of Sciences, 37005, České Budějovice, Czech Republic; Faculty of Science, University of South Bohemia, 37005, České Budějovice, Czech Republic; Department of Aquatic Microbial Ecology, Institute of Hydrobiology, Biology Centre of the Czech Academy of Sciences, 37005, České Budějovice, Czech Republic; Department of Fisheries, Ministry of Natural Resources and Climate Change, 593 Lilongwe, Malawi; Department of Plankton and Microbial Ecology, Leibniz Institute for Freshwater Ecology and Inland Fisheries, (IGB), Alte Fischerhuette 2, D-16775 Neuglobsow, Germany; Institute of Biochemistry and Biology, Potsdam University, Maulbeerallee 2, D-14469 Potsdam, Germany; Cavanilles Institute of Biodiversity and Evolutionary Biology, University of Valencia, E-46980 Paterna, Valencia, Spain; Cavanilles Institute of Biodiversity and Evolutionary Biology, University of Valencia, E-46980 Paterna, Valencia, Spain; Cavanilles Institute of Biodiversity and Evolutionary Biology, University of Valencia, E-46980 Paterna, Valencia, Spain; School of Life Sciences, University of Warwick, CV4 7AL Coventry, United Kingdom; Evolutionary Genomics Group, Departamento de Producción Vegetal y Microbiología, Universidad Miguel, Hernández, 03550, San Juan de Alicante, Alicante, Spain; Water Research Institute, National Research Council (IRSA-CNR), Molecular Ecology Group (MEG), Largo Tonolli 50, Verbania 28922, Italy; Limnological Station, Department of Plant and Microbial Biology, University of Zurich, 8802, Kilchberg, Switzerland; Limnological Station, Department of Plant and Microbial Biology, University of Zurich, 8802, Kilchberg, Switzerland; Limnological Station, Department of Plant and Microbial Biology, University of Zurich, 8802, Kilchberg, Switzerland; Lake and Glacier Ecology Research Group, Department of Ecology, University of Innsbruck, A-6020, Innsbruck, Austria; Lake and Glacier Ecology Research Group, Department of Ecology, University of Innsbruck, A-6020, Innsbruck, Austria; Research Department for Limnology, Mondsee, University of Innsbruck, A-5310, Mondsee, Austria; Research Department for Limnology, Mondsee, University of Innsbruck, A-5310, Mondsee, Austria; Unité d'Ecologie Systématique et Evolution, CNRS, Université Paris-Saclay, AgroParisTech, 91190 Gif-sur-Yvette, France; Unité d'Ecologie Systématique et Evolution, CNRS, Université Paris-Saclay, AgroParisTech, 91190 Gif-sur-Yvette, France; Unité d'Ecologie Systématique et Evolution, CNRS, Université Paris-Saclay, AgroParisTech, 91190 Gif-sur-Yvette, France; Laboratoire Microorganismes: Génome et Environnement, CNRS, Université Clermont Auvergne, 63000 Clermont-Ferrand, France; Laboratoire Microorganismes: Génome et Environnement, CNRS, Université Clermont Auvergne, 63000 Clermont-Ferrand, France; Department of Hydrobiology, Faculty of Biology, Institute of Ecology, Biological and Chemical Research Centre, University of Warsaw, Żwirki i Wigury 101, 02-089 Warsaw, Poland; Department of Hydrobiology, Faculty of Biology, Institute of Ecology, Biological and Chemical Research Centre, University of Warsaw, Żwirki i Wigury 101, 02-089 Warsaw, Poland; Hydrobiological Station, Faculty of Biology, University of Warsaw, Pilchy 5, 12-200 Pisz, Poland; Aquatic Botany and Microbial Ecology Research Group, HUN-REN Balaton Limnological Research Institute, 8237 Tihany, Hungary; Department of Molecular Biology and Biotechnology, Faculty of Biology and Geology, Babeş-Bolyai University, 5-7 Clinicilor Street, 400006 Cluj-Napoca, Romania; Department of Ichthyology and Aquatic Environment, School of Agricultural Sciences, University of Thessaly, 38446 Volos, Greece; Division of Materials Chemistry, Ruđer Bošković Institute, Bijenička Cesta 54, 10000, Zagreb, Croatia; Center of Excellence for Science and Technology-Integration of Mediterranean Region, Zagreb, Croatia; Laboratory of Marine Microbiology, Institute of Oceanography and Fisheries, Šetalište Ivana Meštrovića 63, 21000 Split, Croatia; Department of Freshwater and Marine Ecology, Institute for Biodiversity and Ecosystem Dynamics, University of Amsterdam, Amsterdam 1098 XH, The Netherlands; Leibniz Institute for Baltic Sea Research Warnemünde (IOW), Seestrasse 15, D-18119 Rostock, Germany; Centre for Limnology, Estonian University of Life Sciences, 6117 Vehendi, Tartu County, Estonia; Centre for Limnology, Estonian University of Life Sciences, 6117 Vehendi, Tartu County, Estonia; Department of Aquatic Sciences and Assessment, Swedish University of Agricultural Sciences, 750 07 Uppsala, Sweden; Department of Ecology and Genetics/Limnology, Uppsala University, SE-75236 Uppsala, Sweden; Biological Station Lake Neusiedl, Seevorgelände 1, 7142 Illmitz, Austria; Research and Innovation Centre, Fondazione Edmund Mach, Via E. Mach, 1, 38098 S. Michele all'Adige, Italy; NBFC, National Biodiversity Future Center, 90133 Palermo, Italy; Institute of Microbiology, University of Applied Sciences and Arts of Southern Switzerland, Campus Mendrisio, Via Flora Ruchat-Roncati 15, CH-6850 Mendrisio, Switzerland; Department of Botany and Plant Biology, Microbiology Unit, University of Geneva, Sciences III, CH-1211 Geneva, Switzerland; Institute of Earth Sciences, University of Applied Sciences and Arts of Southern Switzerland, Campus Mendrisio, Via Flora Ruchat-Roncati 15, CH-6850 Mendrisio, Switzerland; Institute of Earth Sciences, University of Applied Sciences and Arts of Southern Switzerland, Campus Mendrisio, Via Flora Ruchat-Roncati 15, CH-6850 Mendrisio, Switzerland; État de Vaud, Direction de l'environnement industriel, urbain et rural (DGE-DIREV), 1066 Epalinges, Switzerland; Department of Functional Ecology, Institute of Botany of the Czech Academy of Sciences, 252 43 Průhonice, Czech Republic; Department of Aquatic Microbial Ecology, Institute of Hydrobiology, Biology Centre of the Czech Academy of Sciences, 37005, České Budějovice, Czech Republic; Department of Aquatic Microbial Ecology, Institute of Hydrobiology, Biology Centre of the Czech Academy of Sciences, 37005, České Budějovice, Czech Republic; Department of Aquatic Microbial Ecology, Institute of Hydrobiology, Biology Centre of the Czech Academy of Sciences, 37005, České Budějovice, Czech Republic; Department of Aquatic Microbial Ecology, Institute of Hydrobiology, Biology Centre of the Czech Academy of Sciences, 37005, České Budějovice, Czech Republic; Department of Aquatic Microbial Ecology, Institute of Hydrobiology, Biology Centre of the Czech Academy of Sciences, 37005, České Budějovice, Czech Republic; Department of Aquatic Microbial Ecology, Institute of Hydrobiology, Biology Centre of the Czech Academy of Sciences, 37005, České Budějovice, Czech Republic; Department of Aquatic Microbial Ecology, Institute of Hydrobiology, Biology Centre of the Czech Academy of Sciences, 37005, České Budějovice, Czech Republic; Faculty of Science, University of South Bohemia, 37005, České Budějovice, Czech Republic; Department of Aquatic Microbial Ecology, Institute of Hydrobiology, Biology Centre of the Czech Academy of Sciences, 37005, České Budějovice, Czech Republic; Department of Aquatic Microbial Ecology, Institute of Hydrobiology, Biology Centre of the Czech Academy of Sciences, 37005, České Budějovice, Czech Republic; Department of Aquatic Microbial Ecology, Institute of Hydrobiology, Biology Centre of the Czech Academy of Sciences, 37005, České Budějovice, Czech Republic; Department of Aquatic Microbial Ecology, Institute of Hydrobiology, Biology Centre of the Czech Academy of Sciences, 37005, České Budějovice, Czech Republic; Faculty of Science, University of South Bohemia, 37005, České Budějovice, Czech Republic; Department of Aquatic Microbial Ecology, Institute of Hydrobiology, Biology Centre of the Czech Academy of Sciences, 37005, České Budějovice, Czech Republic; Department of Aquatic Microbial Ecology, Institute of Hydrobiology, Biology Centre of the Czech Academy of Sciences, 37005, České Budějovice, Czech Republic; Faculty of Science, University of South Bohemia, 37005, České Budějovice, Czech Republic; Department of Aquatic Microbial Ecology, Institute of Hydrobiology, Biology Centre of the Czech Academy of Sciences, 37005, České Budějovice, Czech Republic; Department of Aquatic Microbial Ecology, Institute of Hydrobiology, Biology Centre of the Czech Academy of Sciences, 37005, České Budějovice, Czech Republic; Department of Fisheries Oceanography and Marine Ecology, National Marine Fisheries Research Institute, 81-332 Gdynia, Poland; Department of Aquatic Microbial Ecology, Institute of Hydrobiology, Biology Centre of the Czech Academy of Sciences, 37005, České Budějovice, Czech Republic

**Keywords:** freshwater lakes, microbial food webs, predatory flagellate, Telonemia, CARD-FISH, metagenomics

## Abstract

*Telonemia* are one of the oldest identified marine protists that for most part of their history have been recognized as a distinct *incertae sedis* lineage. Today, their evolutionary proximity to the SAR supergroup (Stramenopiles, Alveolates, and Rhizaria) is firmly established. However, their ecological distribution and importance as a natural predatory flagellate, especially in freshwater food webs, still remain unclear. To unravel the distribution and diversity of the phylum *Telonemia* in freshwater habitats, we examined over a thousand freshwater metagenomes from all over the world. In addition, to directly quantify absolute abundances, we analyzed 407 samples from 97 lakes and reservoirs using Catalyzed Reporter Deposition-Fluorescence *in situ* Hybridization (CARD-FISH). We recovered *Telonemia* 18S rRNA gene sequences from hundreds of metagenomic samples from a wide variety of habitats, indicating a global distribution of this phylum. However, even after this extensive sampling, our phylogenetic analysis did not reveal any new major clades, suggesting current molecular surveys are near to capturing the full diversity within this group. We observed excellent concordance between CARD-FISH analyses and estimates of abundances from metagenomes. Both approaches suggest that *Telonemia* are largely absent from shallow lakes and prefer to inhabit the colder hypolimnion of lakes and reservoirs in the Northern Hemisphere, where they frequently bloom, reaching 10%–20% of the total heterotrophic flagellate population, making them important predatory flagellates in the freshwater food web.

## Introduction

Freshwaters are extremely diverse ecosystems, with a wide variety of trophic states along with substantial dynamics within their complex microbial food webs [1–9]. Bacterivorous protists are critical components of these food webs, estimated to predate upon one-fourth of free-living bacteria every day [10, 11]. It is assumed that the majority of such heterotrophic protists are generally <5 μm (HNF: heterotrophic nanoflagellates), such as the uncultured CRY1 lineage of cryptophytes that is one of the most widespread bacterivore and abundant lineages of HNF in freshwaters [12, 13]. Recent studies have suggested that middle-sized (5–20 μm) HNF are omnivores, feeding not only on bacteria, but also predating upon algae and other microbial eukaryotes [11, 14]. Predatory protists are capable of hunting or immobilizing their prey and ingesting cells of relatively large sizes [15]. Laboratory experiments on microbial food web manipulations with predatory flagellates revealed doubling times comparable to the bacterivorous HNF (i.e. hours to days), and appear ahead of the ciliates in the energy transfer [11]. Some of the known predatory lineages of flagellates are Diplonemea [16, 17], Cercozoa [13, 18], Katablepharida [19], MAST-6 lineage [20], and genus *Telonema* [21]. The predatory role of *Telonema* is mainly described in marine and brackish waters [21] and even though it has been frequently observed in freshwaters, its diversity, distribution, abundance, and ecological role in freshwater microbial communities remain less understood.

More than a century ago, *Telonema* was first described from the marine habitat as a relatively small (6–8 μm long), colorless, elliptical, rigid-bodied flagellate without a contractile vacuole and no close relationship with other known flagellates [22]. A few decades later, another report of *Telonema subtilis* appeared, where it was obtained in culture from brackish waters [23]. Based upon its morphological similarity to the then *Cyathomonas* (now *Goniomonas,* a colourless Cryptophyte), it was decided to place *Telonema* within the family Cyathomonadidae. Subsequently, *T. subtilis* was observed in geographically dispersed marine locations (Arctic, Mediterranean, Japanese coastal waters, etc.) [24, 25] at a wide temperature range (−1°C to 26°C) in both summer and autumn seasons. In general, *Telonema* was found ubiquitously but at low abundances, yet at times accounted for up to 10%–30% of total heterotrophic flagellates. A larger *Telonema* species (diameter 10–20 μm) was observed and provisionally described as *Telonema antarcticum* [26] and found to bloom in summer in an annual study at a bay in Greenland (ca. 200 cells ml^−1^) [27]. *T. antarcticum* was cultured by providing *Rhodomonas* as a food source and its ultrastructure was described in detail [21]. This work [21], along with others [28, 29], described the first molecular phylogenies using 18S rRNA genes and consistently concluded that the sequences appeared quite distinct from any other eukaryotic group, highlighting the still unresolved placement of *Telonema* spp*.* Subsequent ultrastructural analyses combined with molecular phylogenies of 18S rRNA, Hsp90, α, and β-tubulin gene sequences suggested that *Telonemia* represents a deep branching group, placed within its own phylum, i.e. *Telonemia* [30]. The affinity of *Telonemia* derived 18S rRNA gene sequences to Cryptophytes (also speculated before based upon morphological evidence) and Haptophytes was noted, but not considered conclusive. The availability of additional *Telonemia* 18S rRNA gene sequences confirmed previous observations that *Telonemia* represents a widespread phylum and can be grouped into two clades: Group1 with *T. subtilis* and Group2 with *T. antarcticum* [31].

The first indication of freshwater *Telonemia* representatives stems from a microscopic examination of protist samples from Sombre Lake in Antarctica [25] and was later confirmed by sequencing of 18S rRNA gene clone libraries from Lake Pavin [32]. However, molecular phylogenies showed strong support for *Telonemia* being related to Stramenopiles, Alveolates, and Cercozoans, and not to Cryptophytes as was suggested before. Another multigene phylogeny (actin, α-tubulin, β-tubulin, cytosolic HSP70, BIP HSP70, and HSP90) also suggested that telonemids should be grouped together with the SAR supergroup (Stramenopiles, Alveolates, and Rhizaria) [33] rather than with Cryptophytes and Haptophytes. However, a larger phylogenetic analysis with more than a hundred genes recapitulated the *Telonemia* and Cryptophyte grouping [34], and this was retained in a later work combining ultrastructural analyses and multigene phylogenies [35]. These incongruencies were finally resolved with a robust multigene phylogeny, placing *Telonemia* as a sister group to the SAR supergroup, forming the TSAR assemblage (*Telonemia* + SAR) [36].

Multiple additional environmental surveys using amplicon sequencing or clone libraries have repeatedly detected *Telonemia* in a wide variety of habitats, ranging from marine [37–40] to brackish [41] and freshwaters [42, 43], though usually at low abundances. Recently, the dynamics during a spring phytoplankton bloom was reported with the use of a CARD-FISH probe specific for *Telonemia* [44]. A more focused study on both marine and freshwater *Telonemia* reiterated the presence of multiple clades of freshwater *Telonemia*, within the already defined groups Telo-1 and Telo-2, which suggests the possibility of various marine–freshwater transitions within this group [43]. One study also used network analysis of 18S rRNA gene amplicons to show *Telonemia* associated with an unknown ciliate suggestive of predation of *Telonemia* itself [45]. Even more recently, multiple isolates of *Telonemia* have been obtained and new genera have been defined, e.g. *Lateronema*, *Arpakorses*, mostly from marine habitats, but also the first freshwater species: *T. rivulare* has been described [46]. Additionally, a limited number of studies have examined protist communities in rivers using 18S rRNA gene amplicon sequencing in the northern hemisphere including the Saint-Charles, Great Whale, Nelson, and Churchill rivers in Canada [47, 48], the Vistula river in Poland [41], and the Yangtze river in China [49]. These were not focused upon *Telonemia* per se, but have reported the presence of *Telonemia* OTUs, particularly in colder seasons or in brackish regimes.

A systematic examination of the prevalence of *Telonemia* in freshwaters (particularly in lentic habitats), has been missing until now. Moreover, it is unclear if novel, yet undescribed major clades of *Telonemia* thrive in freshwaters. In this work, we used catalyzed reporter deposition fluorescence *in situ* hybridization (CARD-FISH) and a specific probe to directly visualize and quantify *Telonemia* in 97 lakes across Europe, Africa, South America, Australia, and Japan. In addition, we examined its seasonal distribution at four different freshwater sites. Furthermore, we recovered almost 250 18S rRNA gene sequences from >1000 freshwater metagenomes, greatly expanding our knowledge on the ecology of *Telonemia* in freshwaters. Our analyses suggest that *Telonemia* diversity is restricted to the already described main clades (Telo-1 and Telo-2) with a worldwide distribution in freshwater lakes, typically in the cold hypolimnion, and they are largely absent from shallow or hypertrophic water bodies. Based on their ubiquitous presence and occasional peaks of very high abundances in this rather niche, we suggest that *Telonemia* might represent one of the major predatory flagellates of the deep microbial food web in some freshwater lakes.

## Materials and Methods

### Study sites and sampling

Samples for CARD-FISH were collected from 97 freshwater and brackish water habitats covering a broad diversity of trophic states (ultraoligotrophic, oligotrophic, mesotrophic, eutrophic, and dystrophic), continental biomes (e.g. arctic, alpine, continental, Mediterranean, and boreal) at elevations of up to 1921 m asl (Lake Cadagno), and depths of 2–300 m. The sampled habitats spanned across a wide geographical distribution covering five continents (Europe, Asia, Oceania, Africa, and South America). However, we must point out that tropical lakes are underrepresented in our sample collection. For each lake, water samples were collected from the epilimnetic and hypolimnetic layers (except for shallow lakes where samples were collected only from the surface). Several timelines were collected from specific lakes. Four hypertrophic ponds and three dimictic reservoirs of different trophic status in the Czech Republic were studied monthly for six and nine months, respectively. Monthly samples from a monomictic and oligo-mesotrophic lake (Lake Biwa, Japan) were collected for a whole year. Samples were also collected from a temporal high-resolution spring campaign (three times a week) in Řimov reservoir, Czech Republic. In total, we examined 407 CARD-FISH samples from these sites. A complete list of all samples used in this work, along with all physicochemical parameters measured for each sample is provided in Supplementary Table S1.

### Catalyzed reporter deposition-fluorescence *in situ* hybridization

Samples were fixed with formaldehyde (2% final concentration) for up to 24 h at 4°C. About, 30 ml of epilimnetic and 90 ml of hypolimnetic water were filtered on 0.8-μm polycarbonate filters (47 mm), and stored at −20°C for further processing. We used the oligonucleotide probe Telo-1250 (5′ CAGYCAAGGTGGACAACTYGTT 3′) targeting all *Telonemia* [44]. CARD-FISH was performed following the protocol described elsewhere [50] with fluorescein-labeled tyramides. CARD-FISH preparations were analyzed using an epifluorescence microscope (Olympus BX53, Japan) at 1000× magnification. Microscopic images were taken using Zeiss Imager Z2, Carl Zeiss, Oberkochen, DE equipped with a Colibri LED system.

### Preprocessing and assembly of publicly available metagenomic datasets

Adaptor sequences and low-quality bases were removed from the (Illumina) sequences using the bbmap package (http://sourceforge.net/projects/bbmap/). Briefly, the reads were quality trimmed by bbduk.sh (using a Phred quality score of 18). Subsequently, bbduk.sh was used for trimming adapters, and also for the identification/removal of possible PhiX and p-Fosil2 contamination. *De novo* adapter identification with bbmerge.sh was also performed in order to ensure that the datasets meet the quality threshold necessary for assembly. Wherever necessary, metagenomic datasets were assembled independently with MEGAHIT (v1.1.5) (−-k-list 49 69 89 109 129 149) and default settings, otherwise previously available assemblies were used [4, 51–57]. A complete list of all metagenomes used in this work is provided in Supplementary Table S2.

### Retrieval of Telonemia 18S rRNA gene sequences from assembled shotgun metagenomic datasets

*Telonemia* 18S rRNA gene sequences were gathered from previous publications [21, 30, 31, 43, 46]. The metagenomic assemblies were scanned for 18S rRNA gene sequences using ssu-align [58]. All recovered 18S rRNA gene sequences were submitted to the SILVA [59] online classification (https://www.arb-silva.de/aligner/) to identify bonafide *Telonemia* sequences. *Telonemia* 18S rRNA from metagenomic assemblies and published 18S rRNA gene sequences were clustered at 95% nucleotide identity and 100% coverage using cd-hit-est [60]. The representative sequences obtained (*n* = 21) were individually submitted to the IMG/ER service that allows retrieval of related sequences by megablast [61]. All retrieved sequences from IMG/ER were also submitted to the SILVA online classification to confirm that they belonged to *Telonemia*. Finally, only those *Telonemia* sequences with a minimum length of 400 bp were retained for further analysis. A complete table of all sequences (*n* = 771), their sources of origin, and the sequences is provided in Supplementary Table S3.

### CARD-FISH probe specificity

The retrieved sequences were used to test the coverage of probe Telo-1250. After removing sequences that did not have the target region, the alignment of the remaining 476 sequences was imported into the software ARB [62]. As the probe has degenerated bases, all four non-degenerated sequences were tested using the probematch tool. The probe matched 90% of sequences when no mismatches were allowed, and 100% with 0 weighted mismatched option. The probe matched three nontarget sequences retrieved from organisms with distinct morphology (*Symbiodinium*, Syndiniales Group, Cercozoa Novel Clade 2). It is unclear how prevalent Cercozoa Novel Clade 2 is in freshwaters, but it has been observed to be abundant in enrichments from brackish water [18] and the single sequence that matched the probe was retrieved from a coastal margin of the Columbia river [63], which is very different from other sequences in this lineage. Moreover, examination of 18S rRNA gene abundances of these groups in the metagenomes of the same samples, from which CARD-FISH was performed, revealed that most of the time, *Symbiodinium* was <1%. On the other hand, Cercozoa Clade 2 appeared to be quite widespread and abundant in these metagenomes; however, we detected no CARD-FISH signal from *Telonemia* at those sites with high cercozoan abundances (ca. 20%), strongly suggesting that overestimation of *Telonemia* using this probe is negligible (Supplementary Table S4). Moreover, *Telonemia has* larger, pear-shaped cells, a characteristic triangular nucleus, and can be distinguished from flagellates of Cercozoa Novel Clade 2.

### 18S rRNA phylogenetic tree construction

All *Telonemia* 18S rRNA gene sequences retrieved from literature or from metagenomes as described above (minimum sequence length 400 bp) were dereplicated at several nucleotide identity levels (95, 96, 97, 98, 99, and 100%). Alignments were created using mafft-linsi [64] and PASTA [65] at all these dereplication settings and maximum-likelihood phylogenetic trees were constructed using Iqtree2 v.2.2.2.6 31 (settings: -B 1000 --alrt 1000 -m MFP) [66, 67]. The best-fitting evolutionary models were chosen by ModelFinder [68] according to the Bayesian information criterion (BIC). Cryptophyte 18S rRNA gene sequences were used as outgroups for all phylogenetic trees. The delineation of clades Telo-1 and Telo-2 was based upon previous studies [43, 46]. All sequences, alignments, and phylogenetic trees are available at Zenodo (doi: 10.5281/zenodo.11237305). The resulting trees were visualized in iTOL (http://itol.embl.de).

### Quantification of Telonemia 18S rRNA gene sequences in metagenomic datasets

SILVA 138.1 eukaryotic 18S rRNA gene sequences (nr99) were downloaded locally. All *Telonemia* sequences gathered from literature and from locally assembled metagenomes or public servers (IMG/ER) were dereplicated at 99% identity using cd-hit-est [60] and added to the local SILVA 138.1 (nr99) database.

18S rRNA gene sequences were identified in the short-read metagenomes using ssu-align [58]. These short-read eukaryotic metagenomic 18S rRNA sequences were compared using MMseqs2 [69] to the *Telonemia* supplemented nr99 SILVA database (minimum %identity 80, minimum alignment length 100, e-value 1e-5) using a best-hit strategy. 18S rRNA gene sequences originating from organisms known for their extensive rRNA operon presence, such as Dinoflagellata and Ciliophora, were removed before further analysis. Additionally, sequences from multicellular organisms like Metazoa and Embryophyta, as well as those originating from nucleomorphs of Cryptophyceae, were excluded. The results were converted to percentages. The category “others” is a collection of all groups that were either unclassified or < 1% across all datasets.

### Correlation analysis

Spearman correlations between *Telonemia* abundance (CARD-FISH) and environmental parameters were calculated using the R function “cor” [70]. The results of these correlations are provided in Supplementary Table S5.

## Results and Discussion

### Phylogenetic analysis of freshwater Telonemia 18S rRNA

In order to get an impression of the occurrence of *Telonemia* in highly diverse freshwaters, we used a collection of 1027 metagenomic assemblies, in addition to mining assemblies from public databases (see section Materials and Methods, Supplementary Table S1). We recovered 574 *Telonemia* 18S rRNA gene sequences, of which 249 sequences were derived from freshwaters (see section Materials and Methods, Supplementary Table S3). Currently, this represents the largest recovery of *Telonemia* sequences from freshwaters and suggests they are widespread in these habitats but clearly not uniformly distributed.

**Figure 1:**
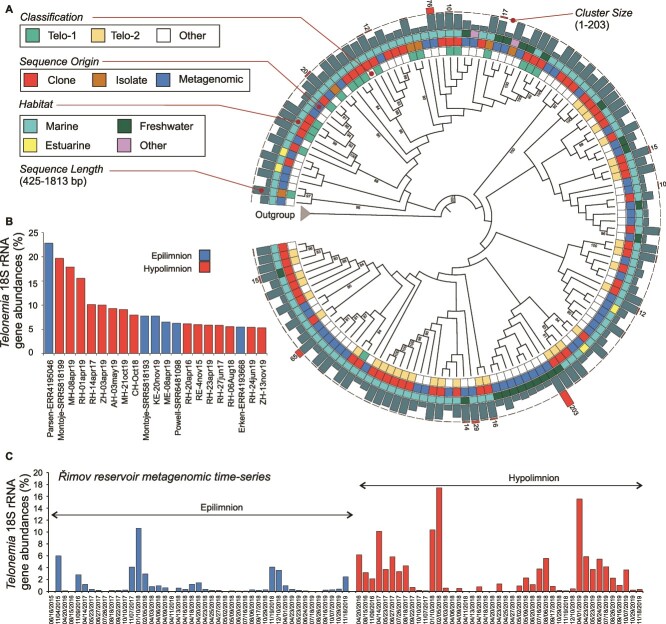
(A) Maximum likelihood phylogenetic tree of representative *Telonemia* 18S rRNA gene sequences clustered at 97% nucleotide identity (shown as a cladogram). The outgroup sequences are not shown but indicated by an arrow. Ultrafast bootstrap values are shown at each node. Two clades, Telo-1 and Telo-2 as defined in previous publications, are shown in different colors. Sequences not classified or <1% are shown as “other.” Isolate sequences are shown in bold. The origin of each sequence (clone library, metagenomic, or isolate) is indicated by colored squares. The length of each sequence, and the number of sequences in each sequence cluster are shown at the right as barcharts. Number of sequences in clusters with >10 sequences is shown (B) *Telonemia* abundances estimated with 18S rRNA gene sequences from metagenomes and (C) in a metagenomic time-series of Řimov reservoir.

Phylogenetic analyses of these recovered sequences with reference sequences from cultured species recapitulated the groupings of Telo-1 (including *T. subtilis*) and Telo-2 (including *T. antarcticum*) as have been obtained before (Fig. 1, Supplementary Figs S1-S4). However, whereas Telo-1 appears to be a well-defined clade in these phylogenetic trees, this is not the case for Telo-2, which has been also earlier described as a polyphyletic clade [46]. Moreover, inferring phylogenetic relationships within *Telonemia* using 18S rRNA gene sequences alone is problematic owing to most clades having low bootstrap support (Fig. 1, Supplementary Figs S1-S4). Using 18S rRNA gene sequences for placing *Telonemia* within the tree of life has largely provided conflicting placements to multiple different groups, which were finally resolved through a phylogenomics approach. It also appears that there is little additional phylogenetic signal within the 18S rRNA sequences themselves for a robust within-phylum clade delineation. The relatively low diversity within the phylum can be illustrated by the recovery of only 17 representative sequences at 95% identity levels. At 96, 97, 98, 99, and 100% identity, we obtained 23, 36, 61, 134, and 430 representative sequences, respectively. No new consistently supported clades were observed. Given the wide diversity of habitats examined here, it is possible that 18S rRNA diversity within this phylum has been exhaustively sampled. We recovered more sequences from the Telo-2 clade (metagenomic: 177 marine, 194 freshwater; clone libraries: 105 marine, 35 freshwater) than for Telo-1 (metagenomic: 69 marine, 66 freshwater; clone libraries: 31 marine, 8 freshwaters), suggesting a generally wider distribution of Telo-2 in freshwaters than Telo-1.

### Abundance of Telonemia in freshwater metagenomes

We examined 589 freshwater metagenomes to obtain a rough estimate of the relative abundances of *Telonemia* in freshwater habitats with respect to other protistan taxa (Supplementary Fig. S5, Supplementary Table S2). Reads of *Telonemia* were found at >1% in 117 samples, >5% in 21 samples, and >10% in six samples (Supplementary Table S2). The highest abundance (ca. 22%) was found in an under-ice sample of Lake Parsens (Sweden). Clustering of 18S rRNA gene taxonomy profiles of samples where *Telonemia* was present did not reveal any outstanding commonalities (Supplementary Fig. S5). These results suggest that *Telonemia* shows a preference for deeper waters as most samples with higher abundances were frequently derived from lake hypolimnia (Fig. 1). This was also supported by a weak, but significant correlation between *Telonemia* percentages by 18S rRNA gene and depth (*n* = 407, Spearman’s *R* = 0.174, *P* value = 4.2e-04). Moreover, examination of longer metagenomic time-series of two sites (Řimov reservoir, Czechia, and Lake Mendota, Wisconsin, USA) showed quite different abundances even though both temperate water bodies are largely eutrophic. Read abundance levels of *Telonemia* 18S rRNA gene sequences in the Lake Mendota dataset were never >1% at any time (Supplementary Table S2). On the other hand, the Řimov reservoir had multiple time points with very high abundances (up to 18%), suggesting *Telonemia* are almost always present in the hypolimnion (Fig. 1). Furthermore, *Telonemia* declined during winter in the hypolimnion, whereas maxima during winter were recorded for the epilimnion. This may also be due in part to the general higher abundance of prey in the epilimnion coupled with the more favorable lower temperatures in winter.

### CARD-FISH analyses of Telonemia in freshwater lakes

**Figure 2:**
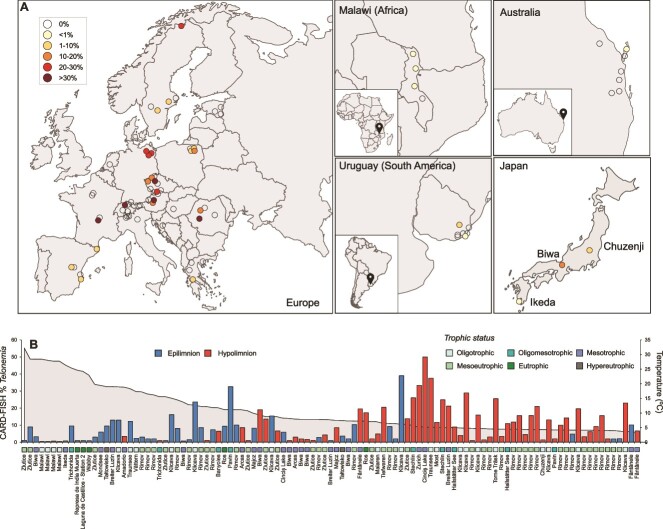
(A) Geographic locations of lake samples used for CARD-FISH in this work. Samples are color-coded according to the maximum %*Telonemia* found at that site using CARD-FISH (see key top left). Sites where *Telonemia* was not detected at all are shown as empty circles. A complete list of all samples is provided in Supplementary Table S1. (B) CARD-FISH counts (%*Telonemia*) in CARD-FISH filters (only those more than 1% are shown here), sorted by decreasing temperature. Epilimnion and hypolimnion samples, along with lake trophic status are shown in different colors.

**Figure 3:**
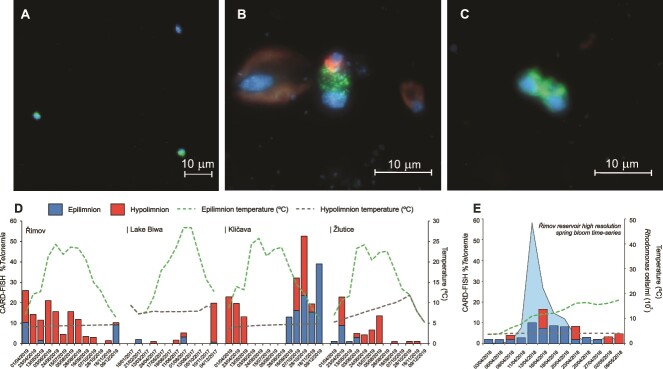
CARD-FISH images of *Telonemia* targeted by the probe Telo-1250. Green: CARD-FISH probe, blue: DAPI, red: autofluorescence (A) from Lake Cinciș (20 m) (B) from Řimov reservoir (0.5 m), *Telonemia* ingesting a *Rhodomonas*. A larger *Cryptomonas* and smaller *Rhodomonas* are also seen left and right, respectively, and (C) a dividing *Telonemia* cell from Breiter Luzin hypolimnion (50 m). All scale bars are 10 μm, panel (A) is in magnification 40X, and panels (B) and (C) are in 100X magnification. (D) Four annual time-series from Řimov reservoir, Lake Biwa, Kličava reservoir and Žlutice reservoir showing relative abundances of *Telonemia* (using CARD-FISH) in epilimnion and hypolimnion. Temperatures are shown as green (epilimnion) and gray-dotted (hypolimnion) lines. (E) Relative abundances of *Telonemia* in epilimnion and hypolimnion during a high-resolution sampling of a spring phytoplankton bloom in Řimov reservoir. Counts of the most abundant Cryptophyte (*Rhodomonas*) are shown as a blue background and temperatures as lines.

CARD-FISH counts of 407 samples confirmed the results already seen in the 18S rRNA gene abundance results (Fig. 1), and *Telonemia* are more abundant in the deeper layers of lakes, where temperatures are generally below 10°C (Fig. 2). In a more extreme case, relative abundances of up to 50% of all eukaryotes were recorded in the hypolimnion of mesotrophic Lake Cinciș (Romania) (Figs 2 and 3, Supplementary Fig. S6). We also did not observe *Telonemia* in lakes from Africa (Lake Malawi), or from Australia and South America (Fig. 2, Supplementary Table S1) likely because of their elevated temperature (>20°C). We found a strong correlation between the abundance assessments (as % *Telonemia*) between CARD-FISH and from metagenomes (*n* = 51, Spearman’s *R* = 0.715, *P* value = 3.84e-09, Supplementary Fig. S7), often not observed for many protist groups [71]. Some possible reasons for concordance may be most likely related to the relatively limited diversity of *Telonemia* 18S rRNA gene sequences, the divergence from other protist groups*,* and the high specificity of the CARD-FISH probe for this group. Additionally, *Telonemia* were completely absent at sampling sites with a maximum depth of ca. 12 m (79 samples) except for two instances where it was still <1% (Supplementary Table S1).

These counts reveal that *Telonemia* are widely distributed in freshwater habitats (in particular deeper ones) at given conditions, but do not reveal any distinct seasonal patterns that are better examined using time-series analyses. To discover such patterns, we conducted monthly sampling for one year from four distinct water bodies. Seasonal profiles of *Telonemia* abundance differed greatly. In Řimov reservoir, *Telonemia* appeared to be present in the hypolimnion throughout the entire year reaching low levels only in the coldest time of the year, concomitantly with maxima in the epilimnion (Fig. 3D). In the Kličava reservoir, *Telonemia* reached maxima of up to 40% of all eukaryotes in the epilimnion during the colder months but could also be simultaneously detected in the hypolimnion (unlike in Řimov). *Telonemia* were completely absent in Kličava in summer and autumn when the reservoir is strongly stratified even though the temperatures in the hypolimnion remained stable around 5°C. In Žlutice reservoir, *Telonemia* almost completely disappeared in the autumn–winter months, and appeared again in spring and further increased in the hypolimnion until autumn. Thereafter, they disappeared from the hypolimnion for almost the entire winter period. Increasing temperatures in summer also coincided with the disappearance of *Telonemia* in the Žlutice reservoir at around 10°C in the epilimnion (Fig. 3D). The hypolimnion in this reservoir is anoxic at this time of the year, which may also explain *Telonemia*’s disappearance there. The lowest abundances, however, were recorded in Lake Biwa, where *Telonemia* were undetected most of the time, or at very low abundances (<5%), but reached a maximum of ca. 20% in a single winter sample. Lake Biwa hypolimnion temperatures appear higher than other lakes (7°C), which is still well below 10°C. Thus, although temperature does appear to be an important factor shaping the occurrence of *Telonemia,* it does not explain entirely the observed patterns in the time series. We also found no correlations with Chl-a, dissolved oxygen, or any other physicochemical parameters (e.g. ammonia, nitrites, nitrates, pH) with relative abundances of *Telonemia* (Supplementary Table S5).

There were several occasions on which *Telonemia* were also found in the epilimnion. On one such occasion, i.e. the spring phytoplankton bloom, they reached up to 15% of all HNF [44]. We analyzed CARD-FISH samples from a high-frequency sampling during the phytoplankton spring bloom (ca. every 3 days) [4] and observed *Telonemia* peaking in the epilimnion (up to 10%) and even ingesting the most abundant Cryptophyte *Rhodomonas* (Fig. 3B). This is likely due to the still relatively low temperatures in the epilimnion in this season and also to the high availability of prey organisms such as cryptophytes. Later in the season, *Telonemia* abundance seems to decrease with increasing temperatures and decreasing abundance of its prey.

Our analyses provide new insights into the distribution and seasonal patterns (at both long and short time intervals) offering clues on possible niche preferences of *Telonemia* in lentic water bodies, which appear to be predicated by a low-temperature regime frequently associated with higher depth (<12 m). It was almost completely absent from shallow or hypertrophic sites. Additionally, *Telonemia* appeared also absent from sites with temperatures >10°C. It also seems that potential prey availability does not appear to be the main driving agent as habitats with extremely high microbial and flagellate populations (e.g. fish ponds) appear totally devoid of *Telonemia* and lake hypolimnia where they are usually resident have lower bacterial and flagellate abundances than surface layers. This is in contrast to the omnipresent occurrence pattern of major bacterivorous flagellates affiliated to the CRY1 lineage, which can be found in a wide temperature range, stretching from the cold hypolimnion to the relatively warmer fish ponds [13]. Indeed, it is likely that *Telonemia* predate upon the CRY1 lineage, which is almost always found in deep hypolimnion at high abundances [13]. The preference of *Telonemia* for deeper water bodies also suggests they are primarily a resident in the hypolimnion, and depending upon favorable environmental conditions (e.g. lower surface temperatures in winter or spring bloom) can transition to the epilimnion. It may also be derived from this that algae like *Rhodomonas* are not their primary prey. However, *Telonemia* show much larger fluctuations in population size, suggesting that even within what appears as a relatively stable hypolimnion, there is sufficient instability in resources through different sedimentation rates, and invasions of additional prey during seasonal algal bloom, thus eventually promoting sudden *Telonemia* blooms (i.e. >20% of all HNF at several sites).

Freshwater food webs, particularly in the hypolimnion, remain little understood. Classical models in ecology have focused largely upon surface layers that show dramatic changes in response to environmental factors [1]. Recently, the distribution and dynamics of protist groups in deeper water layers are increasingly studied revealing a host of diverse flagellates (e.g. kinetoplastids, katablepharids, cercozoans) preying both upon bacteria and other flagellates or smaller algal cells [11, 13, 72]. However, many of these lineages are known solely from sequence data and cultured representatives are scarce (unlike *Telonemia*). This work shows that *Telonemia* are mostly found in cold, deeper freshwaters making them likely significant predatory flagellates in the food web of deep lakes. However, interactions of *Telonemia* flagellates with the larger microbial community still remain obscure. The general approach taken in this work applied to other important and as yet not fully understood lineages in freshwaters will be key to unravel their identities, lifestyles, and dynamics in the largest, yet less studied habitat of deep lakes.

## Supplementary Material

Supplementary_Figure_S1_wrae177

Supplementary_Figure_S2_wrae177

Supplementary_Figure_S3_wrae177

Supplementary_Figure_S4_wrae177

Supplementary_Figure_S5_wrae177

Supplementary_Figure_S6_wrae177

Supplementary_Figure_S7_wrae177

Supplementary_Table_S1_wrae177

Supplementary_Table_S2_wrae177

Supplementary_Table_S3_wrae177

Supplementary_Table_S4_wrae177

Supplementary_Table_S5_wrae177

## Data Availability

All *Telonemia* sequences used in this work, along with all alignments and phylogenetic trees are available in Zenodo (doi: 10.5281/zenodo.11237305). Phylogenetic trees are also available for visualization at https://itol.embl.de/shared/rohitghai.

## The PELAGICS Consortium

Hans-Peter Grossart^4,5^, Antonio Picazo-Mozo^6^, Antonio Camacho^6^, Pedro J. Cabello-Yeves^6,7^, Francisco Rodriguez-Valera^8^, Cristiana Callieri^9^, Adrian-Stefan Andrei^10^, Jakob Pernthaler^10^, Thomas Posch^10^, Albin Alfreider^11^, Ruben Sommaruga^11^, Martin W. Hahn^12^, Bettina Sonntag^12^, Purificación López-García^13^, David Moreira^13^, Ludwig Jardillier^13^, Cécile Lepère^14^, Corinne Biderre-Petit^14^, Anna Bednarska^15^, Mirosław Ślusarczyk^15,16^, Viktor Tóth^17^, Horia L. Banciu^18^, Konstantinos Kormas^19^, Sandi Orlić^20,21^, Danijela Šantić^22^, Gerard Muyzer^23^, Daniel P. R. Herlemann^24,25^, Helen Tammert^25^, Stefan Bertilsson^26^, Silke Langenheder^27^, Thomas Zechmeister^28^, Nico Salmaso^29,30^, Nicola Storelli^31,32^, Camilla Capelli^33^, Fabio Lepori^33,34^, Vojtěch Lanta^35^, Helena Henriques Vieira^1^, Fran Kostanjšek^1^, Kateřina Kabeláčová^1^, Maria-Cecilia Chiriac^1^, Markus Haber^1^, Tanja Shabarova^1^, Clafy Fernandes^1,2^, Pavel Rychtecký^1^, Petr Znachor^1^, Tiberiu Szőke-Nagy^1^, Paul Layoun^1,2^, Hon Lun Wong^1^, Vinicius Silva Kavagutti^1,2^, Paul-Adrian Bulzu^1^,Roudaina Boukheloua^1,2^, Indranil Mukherjee^1^, Hongjae Park^1^, Karel Šimek^1,2^, Vojtěch Kasalický^1^, Michaela M. Salcher^1^, Rohit Ghai^1,*^.

## Notes

Members of The PELAGICS Consortium are listed after the Data availability information.
