# Supplementary material for: Global freshwater distribution of Telonemia protists
Source: ISME J. 2024 Sep 20;18(1):wrae177. doi: 10.1093/ismejo/wrae177 (PMC11512789; doi:10.1093/ismejo/wrae177)
Supplement: Supplementary_Figure_S3_wrae177 [file supplementary_figure_s3_wrae177.pdf]

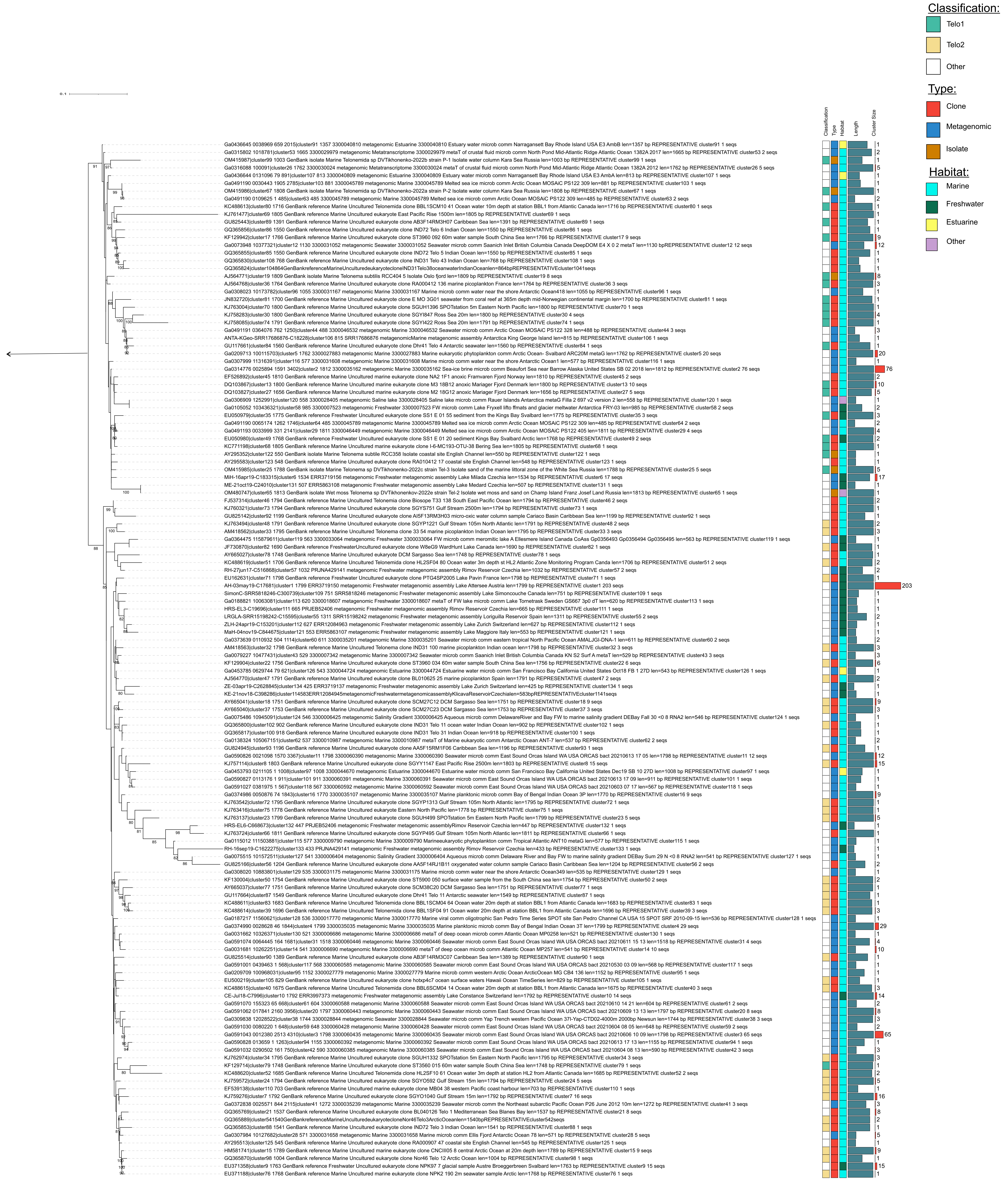

Supplementary Figure S3. Maximum likelihood phylogenetic trees of representative *Telonemia* 18S rRNA gene sequences clustered at 99% nucleotide identity. Alignment was performed using MAFFT. The outgroup sequences are not shown but indicated by an arrow. Tree scale is shown at the left. Ultrafast bootstrap values more than 80 are shown at each node. Telonemia Classification, Type, and Habitat, and size of the cluster of 18s rRNA sequences, and a combined legend are shown to the right.
