# Supplementary material for: Global freshwater distribution of Telonemia protists
Source: ISME J. 2024 Sep 20;18(1):wrae177. doi: 10.1093/ismejo/wrae177 (PMC11512789; doi:10.1093/ismejo/wrae177)
Supplement: Supplementary_Figure_S5_wrae177 [file supplementary_figure_s5_wrae177.pdf]

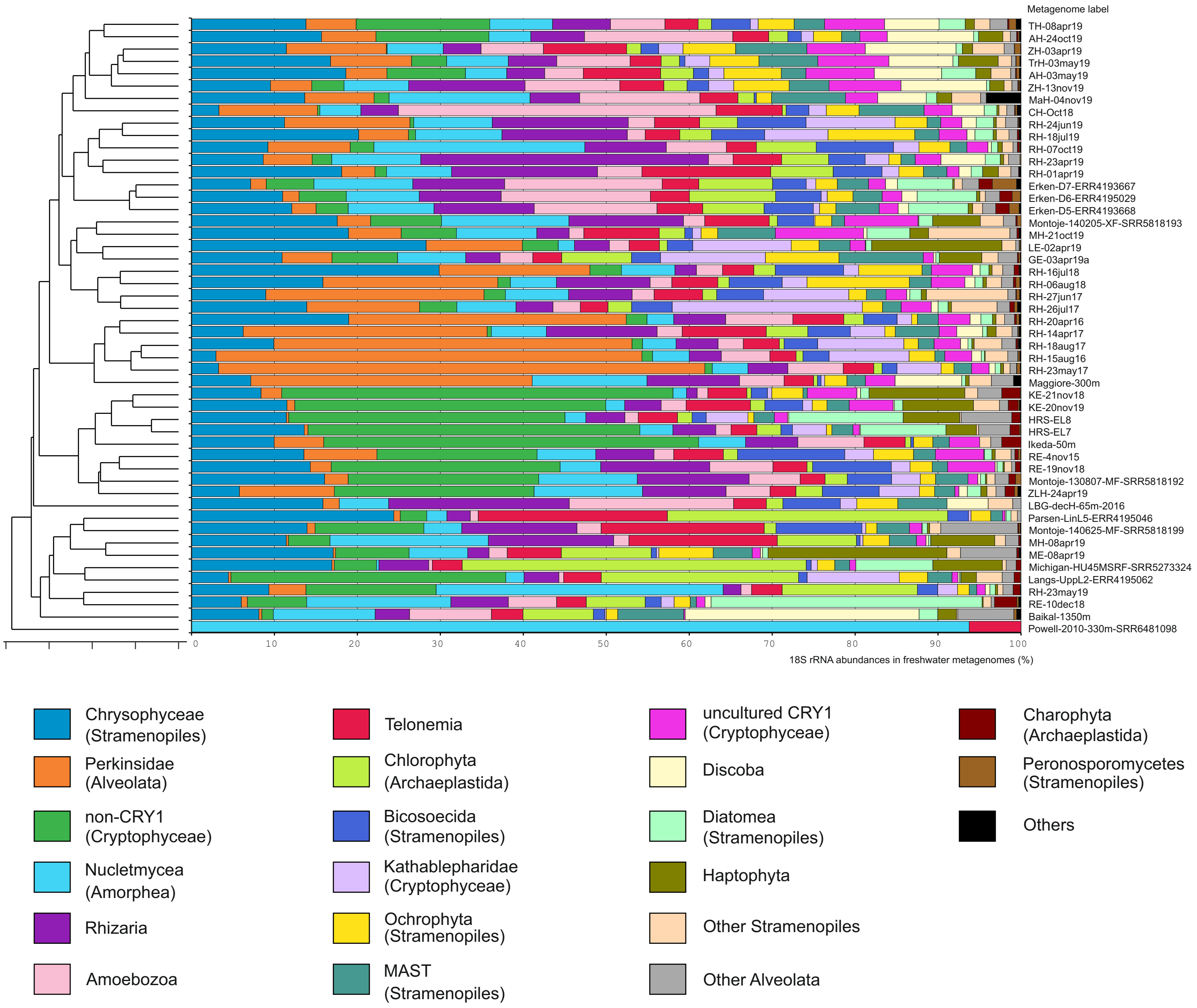

Supplementary Figure S5. Clustering of eukaryal 18S rRNA abundances in freshwater metagenomes. Only those metagenomes with percentage of Telonemia >1% are shown. Datasets are clustered using Bray-Curtis metric and the tree is shown to the left. Sample names are shown to the right. Colors for all top categories are shown below.
