# Supplementary material for: Global freshwater distribution of Telonemia protists
Source: ISME J. 2024 Sep 20;18(1):wrae177. doi: 10.1093/ismejo/wrae177 (PMC11512789; doi:10.1093/ismejo/wrae177)
Supplement: Supplementary_Figure_S6_wrae177 [file supplementary_figure_s6_wrae177.pdf]

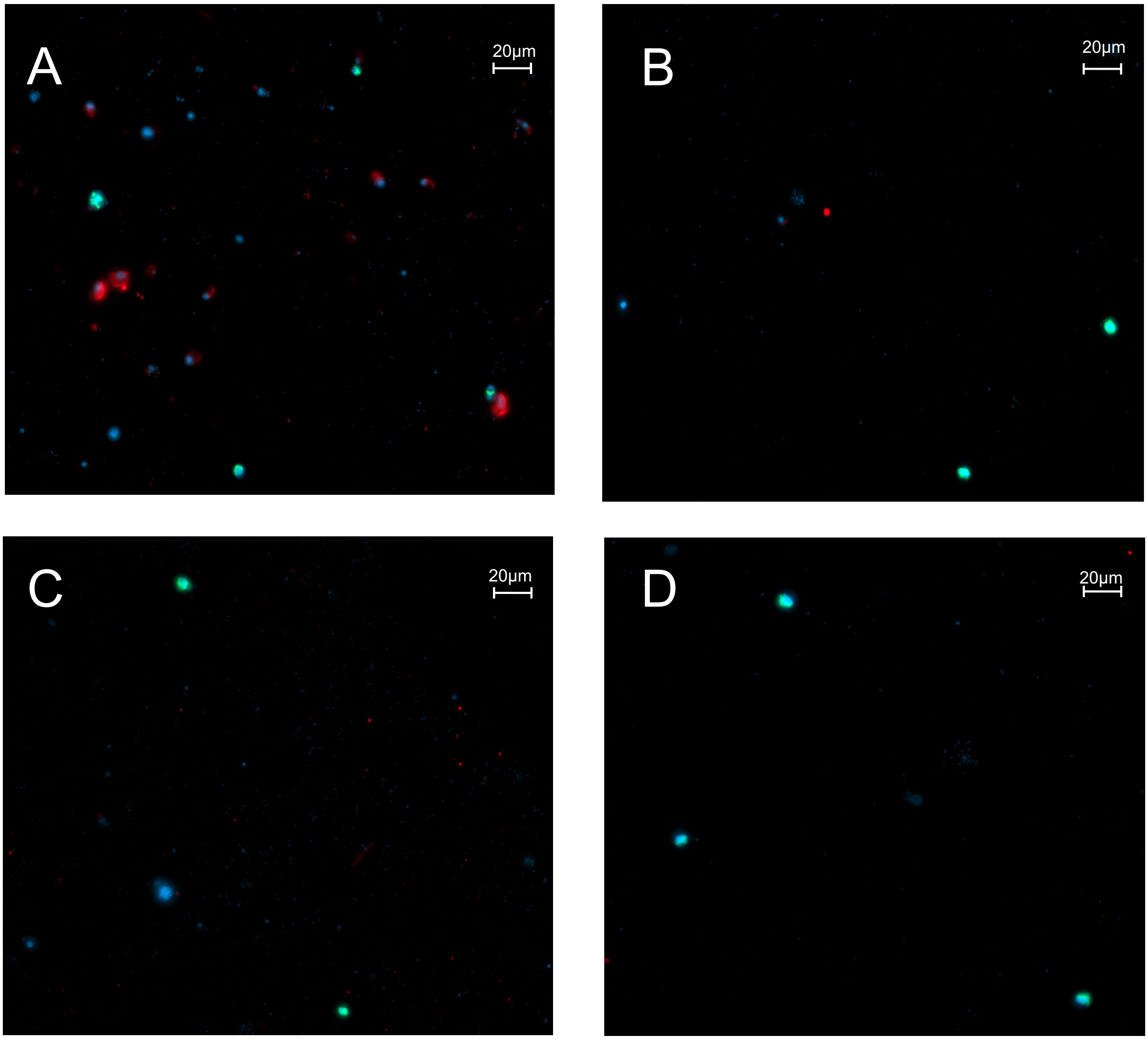

Supplementary Figure S6. CARD-FISH images of *Telonemia* targeted by the probe Telo-1250 (magnification 40X). Green: CARD-FISH probe, blue: DAPI, red: autofluorescence. (A) from Lake Stechlin (40 m), (B) from Lake Breiter Luzin (50 m), (C) and (D) Lake Cinciș (20 m). All scale bars are 20 μm.
