# Supplementary material for: Global freshwater distribution of Telonemia protists
Source: ISME J. 2024 Sep 20;18(1):wrae177. doi: 10.1093/ismejo/wrae177 (PMC11512789; doi:10.1093/ismejo/wrae177)
Supplement: Supplementary_Figure_S7_wrae177 [file supplementary_figure_s7_wrae177.pdf]

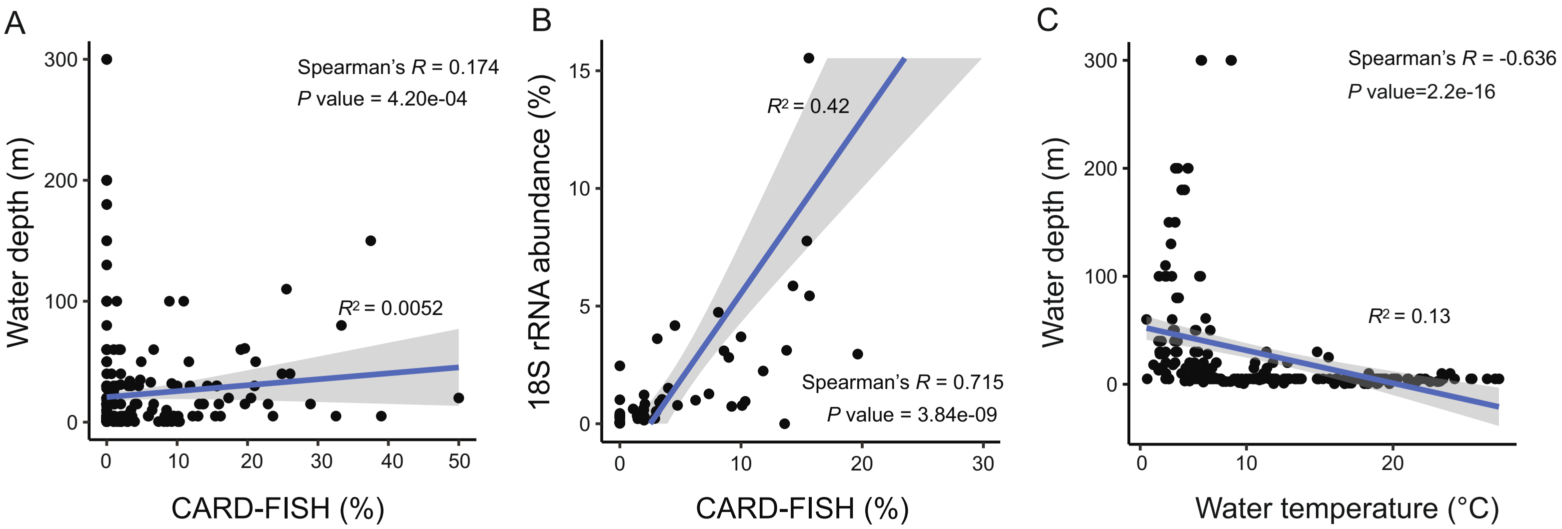

Supplementary Figure S7. Linear regression analysis between (A) CARD-FISH percentage of *Telonemia* and water depth. (B) CARD-FISH percentages of *Telonemia* and *Telonemia* 18S rRNA gene abundance, and (C) water depth and temperature. 95% confidence intervals are shown with the line of best-fit. Spearman's coefficient with *P* value are shown at top-right.
